# Editorial Comment: Anterior component separation technique for abdominal closure in bladder exstrophy repair: Primary results

**DOI:** 10.1590/S1677-5538.IBJU.2022.05.02

**Published:** 2022-08-03

**Authors:** Lisieux Eyer de Jesus, Atila Rondon

**Affiliations:** 1 Universidade Federal Fluminense Hospital Universitário Antônio Pedro Niterói RJ Brasil Hospital Universitário Antônio Pedro, Universidade Federal Fluminense (UFF), Niterói, RJ, Brasil; 2 Hospital dos Servidores do Estado Ministério da Saúde Rio de Janeiro RJ Brasil Hospital dos Servidores do Estado, Ministério da Saúde; Rio de Janeiro, RJ, Brasil; 3 Universidade do Estado do Rio de Janeiro Hospital Universitário Pedro Ernesto Rio de Janeiro RJ Brasil Hospital Universitário Pedro Ernesto. Universidade do Estado do Rio de Janeiro (UERJ), Rio de Janeiro, RJ, Brasil; 4 Hospital Federal Cardoso Fontes Ministério da Saúde Rio de Janeiro RJ Brasil Hospital Federal Cardoso Fontes, Ministério da Saúde, Rio de Janeiro, RJ, Brasil

Francisco Nicanor Araruna Macedo^1^, Eduardo Corrêa Costa^2^, Jovelino Quintino de Souza Leão^3^, Antônio Carlos Amarante^4^, Fernanda Ghilardi Leão^5^, Hélio Buson Filho^6^, et al.

J Pediatr Urol. 2022 Apr 22;S1477-5131(22)00159-0.

## COMMENT

Kelly´s Together Project (KTP) is a great initiative. The results of this work may bring very important information to the Pediatric Urologists community.

The opportunity to treat a relatively high number of OIES cases, including complex patients with a single and standardized technique is unique. The number of patients tends to be high because of Brazilian´s territorial scope and big population, where legal abortion is prohibited. The availability of the National Brazilian Health Service facilitates logistics.

KTP is similar to other programs to treat bladder exstrophy going on in India and North America, using other techniques. In due time it may be possible to compare results between big cohorts treated with different techniques at the same time frame.

The idea of professional consortia is a great development in what concerns rare surgical diseases, and the recent availability of online transmission of surgeries in real-time time and of planning online meetings adds to its implementation. Financial support from the government, in the case of countries counting on public health systems, is needed for travel expenses and to allow paid licenses for the professionals to participate in the initiative.

The present paper (1) is the first to be presented by KTP group and deals with one of the most difficult aspects of exstrophy treated without pelvic osteotomy: the closure of the abdomen, especially after the first days of life and in cases exhibiting large pubic diastasis. The authors ingeniously associate techniques of rectus muscle releasing and myofascial compartment mobilization. We wonder whether associating intramuscular pre-operative botox injections would also help (2). This would be easy to accomplish, as surgeries are usually programmed electively after 3 months of age.

The results described by the authors are great but must be evaluated with a grain of salt, as long-term results are as yet unknown, especially in what concerns genital prolapse in females, late sexual results, and the possibility of dyspareunia and/or penile fracture attaining the released penises, that are fixed on myofascial structures and will not count on the physiologically normal long osseous anchorage of the corpora during coitus in adulthood. To the best of our knowledge, those data are not yet available, either concerning the “classical” Kelly technique (that used radical tissue mobilization to treat the penis and bladder neck AFTER neonatal bladder closure (3)) or the new adaptations being used now, that propose radical tissue mobilization to close the bladder and bladder neck and penile reconstruction simultaneously.

Mr. Justin Kelly has recently suggested that the right term to describe the surgical treatment of bladder exstrophy is to construct, not to reconstruct, as to reconstruct means to put up something together again, while to construct means creating something new (4). In OIES cases the surgeon does not “recover” tissues. He/she build something new from scratch, using abnormal tissues. This applies to all techniques described at the moment and probably reflects on the results of any technique.

The authors are to be congratulated for their persistence, resilience, and personal investment in making clinical research. Their effort is at the same time valuable, stimulating and beautiful. This paper is the first from their group, but, for sure, many more will follow.
